# Supplementary material for: Genome-Wide DNA Methylation Profiling Solves Uncertainty in Classifying NSD1 Variants
Source: Genes (Basel). 2022 Nov 19;13(11):2163. doi: 10.3390/genes13112163 (PMC9690023; doi:10.3390/genes13112163)
Supplement: Supplementary file 1 [file genes-13-02163-s001.zip › genes-2021550-supplementary.pdf]

SUPPLEMENTAL DATA

Clinical case reports

S7 (NM_022455.2: c.5418G>T; p.Trp1806Cys)
The patient, a male, was born with auxological parameters within the normal range. No prenatal data were available. At our first observation (2 years) he presented with macrocrania (97th centile) and macrosomia (both weight and height at 90th centile), hypotonia with hyperlaxity, psychomotor development delay, language delay, prominent forehead, deep set eyes, large ears, and pointed chin. A brain MRI revealed mild dilatation and asymmetry of lateral ventricles, mild non-specific periventricular hyperintensities and mild frontotemporal enlargement of subarachnoid spaces. He also showed posterior corpus callosum hypoplasia and mild hypoplasia of the right temporal lobe. At a subsequent observation (11 years), he presented with mild ID, thoracolumbar scoliosis treated with a corset, limb asymmetry, weight was 42.5 Kg (75th centile), height 159.5 cm (97th centile) and OFC 58.5 cm (>97th centile). At our last examination (14 years) cognitive assessment was performed (total IQ: 86, WISC-IV).

S8 (NM_022455.2: c.4857T>G; p.Cys1619Trp)
The patient, a female, is the second child of healthy non-consanguineous parents. The proband was born by cesarean section at the 37th week after an uneventful pregnancy. Birth weight was 2,720 g (25th centile), length 48 cm (50th centile), head circumference 34 cm (50th centile). Apgar scores were 7 and 8 at 1 and 5 minutes, respectively. Clinical evaluation showed tall stature, hypotonia, mild developmental delay, advanced bone age (+2 years), dolichocephaly, high forehead, down-slanting palpebral fissures, dysmorphic ears, and triangular pointed chin. Two-Dimensional color-Doppler echocardiography showed pulmonary valve stenosis and muscular ventricular septal defect. Cerebral MRI, EEG, abdominal ultrasound, ophthalmological and audiological evaluations were normal.

S9 (NM_022455.2: c.6454C>G; p.Arg2152Gly)
The patient, a female, is the unique child of healthy non-consanguineous parents. Family history was unremarkable. She was born by Cesarean section at the 38th week after an uneventful pregnancy obtained by ART. Birth weight was 3,100 g (50th centile), length 52 cm (75th centile), head circumference 37 cm (75th centile). Apgar scores were 9 and 10 at 1 and 5 minutes, respectively. Clinical evaluation showed speech delay, mild learning difficulties, tall stature, advanced bone age (+18 months), macrocephaly, triangular face, high forehead, downslanting palpebral fissures, deep set eyes, prominent upper central incisors. Cerebral MRI, EEG, two-Dimensional color-Doppler echocardiography, abdominal ultrasound, ophthalmological and audiological evaluations were normal.

S10 (NM_022455.2: c.4786T>C; p.Cys1596Arg)
The proband, a male, is the second child of healthy non-consanguineous Italian parents. Family history was unremarkable. He was born at term by Cesarean section. The pregnancy was complicated by maternal insulin-dependent diabetes. Birth weight was 4,220 g (97th centile, +1.87 SD); length 54.5 cm (>97th centile, +2.39 SD) and OFC 37.5 cm (>97th centile, +2.35 SD). Apgar scores was not available. Perinatal period was complicated by hypotonia, respiratory distress and hypoglycemia. Transfontanellar ultrasound showed periventricular hyperechogenicity with abnormal visualization of corpus callosum and partial agenesis of septum pellucidum. Brain MRI also disclosed ventricular enlargement, enlargement of the subarachnoid spaces, persistence of cavum septi pellucidi and cavum vergae. Developmental milestones were delayed (he walked alone at 18 months, first words at 9 years).
Clinical evaluation at 26 months disclosed macrosomia (weight 20 kg, >97th centile; height 108 cm, >97th centile, and OFC 53.3 cm, >97th centile), long triangular face, high frontal hairline, mild epicanthus, low set ears, narrow palate and pointed chin suggestive of Sotos syndrome. Levoscoliosis with lumbar lordosis and a patent forame ovale were also noted. Bone age evaluation revealed an advanced ossification of approximately 12 months. A CGH-array exam revealed a small *de novo* 16p13.3 duplication classified as variant of uncertain significance.

S11 (NM_022455.2: c.6128T>C; p.Phe2043Ser)
The proband, a male, is the third child of healthy non-consanguineous Italian parents. He was born at term after an uneventful pregnancy by Cesarean section because of fetal distress. Birth weight was 4,100 (86th centile), length 52 cm (76th centile) and OFC 38 cm (90th centile). He presented with severe axial hypotonia, scoliosis, gastro-esophageal reflux and drooling, repeated apnea episodes and atrial septal defect ostium secundum type. At our first clinical evaluation (1 month), distinct Sotos syndrome facial features were observed: macrocrania with prominent and high forehead, dolichocephaly, downslanted palpebral fissures, hypertelorism, bushy eyebrows with lateral sparsening, long face with triangular and pointed chin. He presented with developmental delay and moderate hypotonia, feet oedema, cutis laxa abundantly redundant. A cerebral MRI showed bilateral mild dilatation of temporal and frontal sulci. At our subsequent observation (11 months), height was 83 cm (99th centile) weight 13,800 gr (>97th centile) OFC 52 cm (97th centile). At 1 year and 10 months, he presented with seizures pharmacologically treated with carbamazepin. Developmental delay was noticed. He walked autonomously at 16 months and presented a moderate speech delay, with few words spoken.

S12 (NM_022455.2:c.4917_4925delCTGTATAAC; p.Cys1640_Thr1642del)
The proband, a female, is the second child of healthy non-consanguineous Italian parents. Family history was characterized by recurrent miscarriage. She was born at term by Cesarean section. The pregnancy was complicated by polyhydramnios and maternal insulin-dependent diabetes. Birth weight was 4,310 g (>90th centile, +2.72 SD) and OFC 39 cm (>97th centile, +4.49 SD). Length and Apgar scores were not available. Perinatal period was complicated by neonatal hypoglycemia. Developmental milestones were delayed (he walked alone at 24 months with ataxic gait, first words at 9 years).
Clinical evaluation at 18 months disclosed macrosomia (weight 14 kg, >97th centile; height 92 cm, >97th centile; OFC 52.6 cm, >97th centile). Clinical evaluation at 27 months showed frontal bossing, high frontal hairline, telecanthus, and pointed chin. Bone age was advanced of approximately 3 years. Brain MRI disclosed ventricular enlargement, corpus callosum hypoplasia, frontotemporal enlargement of subarachnoid spaces, and enlarged cisterna magna. A CGH-array were normal.

S13 (NM_022455.2: c.6371G>C; p.Cys2124 Ser)
The proband, a female, was born at 39 gestational weeks from healthy non-consanguineous parents. The pregnancy was complicated by polyhydramnios and maternal hypertension. A prenatal karyotype was unremarkable. Her birth weight was 4,060 g (>97th centile) and length 53 cm (>97th centile). Subsequent serial growth, height-weight and occipitofrontal circumference measurements were always above the reference centiles for age. She had mild developmental delay with specific involvement of language: first words were pronounced at 1 year, while the subsequent linguistic production was achieved after 3 years. Cognitive assessment disclosed a total IQ of 74. The abdominal ultrasound examination showed a slight renal asymmetry with left kidney > right kidney of about 1 cm. Bone age was advanced of about 1 year. An echocardiogram disclosed a mitral valve prolapse.

S14 (NM_022455.2: c.6215G>C; p.Cys2072Ser)
The proband, a female, is the first child of healthy non-consanguineous Italian parents. She was born at term after an uneventful pregnancy by cesarean section because of fetal distress. Apgar scores were 5 and 8 at one and five minutes. Length 51 (71th centile), weight 3,620 (66th centile) and OFC 36 cm (75th centile). She presented with bronchopulmonary dysplasia, axial hypotonia, drooling and atrial septal defect ostium secundum type. She also showed prolonged jaundice, suction difficulties and stipsis. At our clinical evaluation (7 months), Sotos syndrome facial features were observed, including macrocrania with prominent and high forehead, dolichocephaly, downslanting palpebral fissures, hypertelorism, long face with triangular and pointed chin. Height was 73 cm (99th centile), weight 8,150 gr (79th centile), and OFC 44 cm (88th centile). She presented with developmental delay and moderate hypotonia. At 1 year, MRI showed mild dilated ventricles, wide aspect of the periencephalic spaces and thin corpus callosum. She also was

diagnosed with moderate roto-scoliosis and kyphosis, treated with a corset. At last observation (14 months), height was 87 cm (>99th centile), weight 12,900 gr (99th centile), and OFC 47 cm (86th centile).

S15 (NM_022455.2: c.1096G>A; p.Val366Met)
The proband, a girl was born at term after an uneventful pregnancy with length and weight at 75th centile, and OFC >97th centile. She had normal psychomotor development but presented with seizures and EEG anomalies. At 7 years, she presented with macrocrania (OFC 55.5 cm, >97th centile), prominent forehead, short palpebral fissures, deep set eyes, short philtrum, everted lower lip, fetal pads, short first finger and hallux, bilaterally. Brain MRI was normal.

S16 (NM_022455.2:c.7393C>T; p.Gln2465*)
The proband, a female, is the first child of healthy non-consanguineous Italian parents. Family history was unremarkable. The pregnancy was complicated by meconial peritonitis at the 20th week of gestation. Following prenatal genetic analysis, the parents were found to be healthy carriers of delta F508 mutation in *CFTR* and the fetus was suspected to be affected by cystic fibrosis. The diagnosis was confirmed by a postnatal test (homozygosity for delta F508). Birth weight was 3.58 kg (63th centile), and OFC was 35 cm (54th centile). At birth, the girl presented dysmorphic features (relative macrocrania, short neck, and hypertelorism). On the first day of life, the proband underwent abdominal drainage placement, after tracheal intubation and mechanical ventilation for respiratory insufficiency due to abdominal overdistension. She underwent exploratory laparotomy surgery for a large meconium cyst, ileal perforation, and ileal atresia. For this reason, she underwent intestinal resection (about 4 cm) with end-to-end anastomosis. At 1 month of age, the proband showed poor spontaneous motility, relative macrocrania, flat occiput, body disharmony, high forehead with bulging, sparse eyebrows, flat nose root, micro-retrognathia, pinnae anomaly with folded and indented lobule with brachycephalic conformation and short limbs. The brain, cardiac and abdominal ultrasound assessment as well as eye examination were normal. An array-CGH analysis disclosed a heterozygous deletion at Xq21.1, which was inherited from the mother.

S17 (NM_022455.2:c.1782T>C; p.(Pro594=)
The patient, a male, is the unique child of non-consanguineous parents. The proband was born by Cesarean section at the 41th week of an uneventful pregnancy. Birth weight was 4,370 g (>97th centile), length 54 cm (>97th centile), head circumference 38.8 cm (>97th centile). Apgar scores were 8 and 9 at one and five minutes. Clinical evaluation showed mild developmental delay, epilepsy (treated with valproic acid), tall stature, advanced bone age (+18 months), scoliosis, macro/dolichocephaly, high forehead, down-slanting palpebral fissures, triangular pointed chin. Brain MRI, two-Dimensional color-Doppler echocardiography, abdominal ultrasound, ophthalmological and audiological evaluations were normal.
His mother has macro/dolichocephaly (head circumference 59 cm), tall stature (height 181 cm), and reported mild learning difficulties at school.
